# Evaluation of the Proximal Ulna Dorsal Angulation for Ulnar Component Sizing in Elbow Prosthetic Reconstruction After Distal Humeral Resection of Tumor

**DOI:** 10.5435/JAAOSGlobal-D-20-00062

**Published:** 2020-05-19

**Authors:** Caleb M. Yeung, Jonathan Lans, Joseph B. Kuechle, Zachary Wright, Connie Y. Chang, Santiago A. Lozano-Calderón

**Affiliations:** From the Orthopaedic Oncology Service, Department of Orthopaedic Surgery, Massachusetts General Hospital, Harvard Medical School, Boston, MA (Dr. Yeung, Dr. Lans, Dr. Kuechle, Wright, and Dr. Lozano-Calderón), and the Division of Musculoskeletal Radiology, Department of Radiology, Massachusetts General Hospital, Harvard Medical School, Boston, MA (Dr. Chang).

## Abstract

**Introduction:**

Elbow prosthetic reconstruction after distal humeral tumor resection is challenging. We identify the value of the proximal ulna dorsal angulation (PUDA) as an easily-measured radiographic parameter that can help inform ulnar component sizing in the Solar Elbow System (SES) and the Modular Universal Tumor and Revision System (MUTARS), two modular prosthetic systems that are commonly used after tumor resection in this anatomic location. We hypothesized that a larger PUDA measurement would require smaller ulnar stems.

**Methods:**

Demographic data and PUDA measurements were retrospectively reviewed for 514 patients. Multivariate regression was used to determine the effects of patient demographic data on the PUDA. PUDA measurements were collected by three independent reviewers on lateral elbow radiographs. MUTARS and SES templating software was then used to validate the relationship between the PUDA and ulnar stem sizing.

**Results:**

Regression analysis showed no substantial contribution of demographic variables to the PUDA measurement (adjusted R2 = 0.02, F(6, 508) = 2.704, *P* = 0.01). The MUTARS implant fit 97% of elbows with a PUDA <5° and 91.6% of elbows with PUDA ≥5° (*P* = 0.26). The largest SES combination fit 100% of elbows with a PUDA ≤10° versus 93% of elbows with a PUDA >10° (*P* = 0.029). Elbows accommodating the largest SES combination had a smaller median PUDA (5.4° versus 11.7°, *P* = 0.034); elbows accommodating the MUTARS implant had a smaller median PUDA (5.4° versus 5.8°, *P* = 0.34).

**Discussion:**

The PUDA is a valuable and easily used preoperative planning tool for prosthetic elbow reconstruction after tumor resection. The proximal ulna dorsal angulation can be easily measured to predict ulnar component fit and reduce intraoperative complications. In patients with a PUDA ≥5°, ulnar component stem fit for current systems may be more challenging.

The elbow is a challenging site for reconstruction in oncology patients after tumor resection in the distal humerus.^1^ Total elbow arthroplasty for reconstruction of the distal humerus after tumor resection is often complicated by infection, prosthetic loosening, and osteolysis.^2,3^ Given the complexity of reconstruction at this site, modular prosthetic systems are used instead of more common conventional total elbow systems or other reconstruction mechanisms such osteoarticular allografts.^4^ Commonly used modular total elbow systems for reconstruction that also have available templating software for surgeon use are the Solar Elbow System (SES) by Stryker and the Modular Universal Tumor and Revision System (MUTARS) by Implantcast GmbH. Both these systems require preparation of the ulnar canal into which an ulnar stem component is placed. This requires the selection of an appropriately sized ulnar stem component to prevent perforation of the component through the dorsal cortex of the ulna.

Although the anthropometric information of other upper extremity joints such as the shoulder and wrist has been well studied, there is a lack of data available concerning the osseous anatomy of the proximal ulna for preoperative planning and prosthetic design of elbow reconstructive systems.^5,6,7,8^ More information concerning meaningful predictive anatomic parameters of this location could help reduce perioperative complications such as perforation of the dorsal cortex of the ulna, periarticular fracture, or stem component failure. Previous studies investigating the osseous anatomy of the ulna have demonstrated that the ulnar cortex is relatively thin, and that overestimation of the minimal canal size of the ulna can occur even with routine frontal and lateral radiographs.^9^

In this study, we use the proximal ulna dorsal angulation (PUDA), defined as the angle formed by the intersection of lines drawn parallel to the nonarticulating outer cortex of the olecranon and the dorsal diaphyseal cortex of the ulna (Figure 1, A), as a predictive factor for the size of the ulnar stem component in preoperative templating. The PUDA has previously been measured with averages of 5.7^o^ and 4.5^o^ in previous smaller series of 100 and 54 measurements, and was found to be a reliable measurement with good intraobserver and interobserver reliability.^10,11^ We aimed to validate these findings and hypothesized that a larger PUDA measurement would necessitate a smaller stem size for the ulnar component, as larger stems in this patient group could compromise the integrity of either the dorsal ulnar cortex or the triceps tendon attachment.

**Figure 1:**
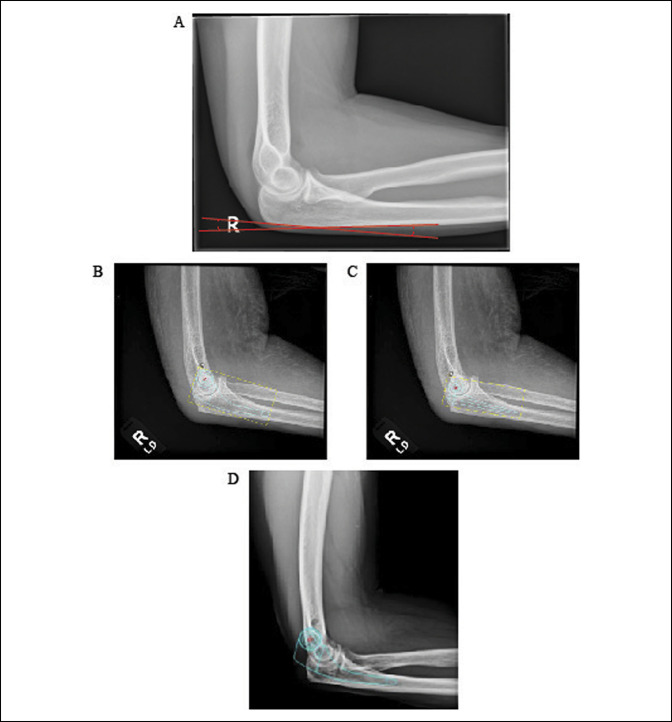
**A**, Measurement of the proximal ulnar dorsal angulation. **B**, Templated MUTARS total elbow implant. **C**, Templated SES total elbow implant. **D**, Template showing a MUTARS total elbow implant which does not fit the elbow given the large PUDA measurement. MUTRS = Modular Universal Tumor and Revision System, PUDA = proximal ulna dorsal angulation, SES = Solar Elbow System

The goal of our study is to inform preoperative planning for ulnar component stem sizing to maximize component fit while limiting complications in elbow prosthetic reconstruction. In this study, we evaluate the normal variation of the PUDA in the general cohort in the largest known series in the literature and also evaluate demographic predictors of the PUDA using multivariate regression analysis. We also evaluate the effect of the PUDA on the sizing of the ulnar stem component in SES and MUTARS templating.

## Methods

This study was a retrospective, Institutional Review Board–approved study consisting of 514 patients from our tertiary referral institution. Patient records and 514 lateral elbow radiographs were reviewed over a period of 6 months; radiographs were all performed by the Department of Radiology of our institution. Using the Agfa ICIS PACS program, the electronic medical record (EMR) was reviewed for radiographs matching the search criteria of a lateral elbow radiograph obtained between a 1-year period in a patient of at least 18 years of age at the time of the radiograph.

These radiographs were reviewed first for determination of inclusion or exclusion from the study. Inclusion criteria were (1) either a native elbow (considered to be a normal radiograph) or a native ulna without any pathology or previous fracture or reconstruction (radius or humerus may have had the presence of fracture, implant, or other previous surgical intervention or reconstruction; these were considered to be a “pseudonormal” radiograph) and (2) age greater than or equal to 18 years. Exclusion criteria were (1) the presence of any pathology, previous fracture, or surgical intervention or reconstruction of the ulna; (2) age less than 18 years; (3) technically inadequate lateral view of the elbow on radiograph as determined by study personnel; and (4) incompletely recorded demographic data.

After inclusion and exclusion criteria were applied to flagged radiographs satisfying our earlier search criteria, the variables of body mass index (BMI), height, weight, age, sex, and race were determined from the EMR. Race as recorded in our EMR consisted of white, black, Hispanic, Asian, other, declined, or unknown.

PUDA measurements were conducted by three separate individuals twice each. More than three months of time were allowed to elapse between initial and repeat measurements by each observer to further minimize chances of recall bias.

Six age brackets were created before analysis: ages 18 to 40, 41 to 50, 51 to 60, 61 to 70, 71 to 80, and 81 and older. A pre hoc power analysis was conducted to satisfy a Pearson's correlation coefficient of 0.30 between the PUDA and the variables of interest including age bracket, sex, race, and BMI. Using this, we determined that a total of 85 patients per age bracket, or a total of at least 425 patients, was required for a power of 80% and a 2-sided *p*-value of 0.05. To account for the possibility of incompletely recorded demographic data that would require exclusion of a record, we collected more patients per each age bracket with a total of 514 patients, with at least 85 patients per age bracket.

Patients were stratified into PUDA groups of <5°, 5 to 10°, and >10°; templating of MUTARS and SES was then done using randomly selected lateral elbow radiographs of 221 patients (with at least 30 represented from each angle group). Although all 514 elbows were initially templated, we noted a disproportionately low number of PUDA measurements satisfying the >10° category. To minimize confounding of any potential effect due to unequal numbers between groups, we templated the maximum number of elbow radiographs possible for each angle category that allowed for preservation of the observed effect during the templating process. Calibration of radiographic images was done based on pixels/mm of the original radiograph.

Templating was done using the TraumaCad software system and modified to satisfy dimensions and locations (Figure 1, B–D). The MUTARS has one size and a curved ulnar stem. The SES has three straight ulnar stem lengths (63, 53, and 50 mm) and small, standard and large sizes. There were 2 patients in which the large SES with a 63-mm ulnar stem did not fit; therefore, we stratified the SES into 2 groups: (1) large size and a 63-mm stem, and (2) other smaller combinations.

For statistical analyses, intra-rater and inter-rater reliability was determined using one-way and two-way random consistency analysis of Cronbach's alpha, respectively. Multivariate regression was used to determine the effects of age, sex, race, height, weight, and BMI on PUDA (Table 1). Dummy numerical values were assigned in the cases of sex and race to facilitate our logistical regression. The Fisher exact test and the Mann-Whitney *U* test were used to determine the effect of the PUDA on ulnar stem selection using the PUDA as a categorical and continuous variable, respectively. *P* < 0.05 was considered significant for all tests. PUDA measurements did not satisfy the Shapiro-Wilk test for normality. Thus, the median values for the PUDA as well as the values for the first and third quartiles are reported herein.

**Table 1:** Descriptive Characteristics and the Proximal Ulna Dorsal Angulation Across Age Groups

Age Group	Average Height (m)	Average Weight (kg)	Average BMI (kg/m^2^)	Mean PUDA (°) (95% CI)	SD PUDA (°)	Mean PUDA (°) (Q1, Q3)
Younger than 40	1.70	79.38	27.23	4.41 (3.71-4.57)	2.06	3.97 (1.9, 6.15)
41-50	1.67	80.74	28.32	4.80 (4.31-5.29)	2.32	5.10 (3, 6.5)
51-60	1.67	85.91	29.91	4.60 (4.10-5.10)	2.38	4.63 (3, 6.3)
61-70	1.63	77.93	28.74	4.43 (3.89-4.97)	2.54	4.58 (2.4, 6.2)
71-80	1.67	81.74	29.34	5.22 (4.71-5.73)	2.48	5.10 (3.65, 6.9)
Older than 80	1.62	70.04	26.52	5.17 (4.58-5.75)	2.85	5.23 (3.5, 6.85)

BMI = body mass index, CI = confidence Interval, PUDA = proximal ulna dorsal angulation

## Results

For patients younger than 40 years, the median PUDA was 4.3° (Q1 = 1.9°, Q3 = 6.15°); for 41 to 50 years, 5.2° (3°, 6.5°); 51 to 60 years, 4.5° (3°, 6.3°); 61 to 70 years, 4.6° (2.4°, 6.2°); 71 to 80 years, 5.1° (3.65°, 6.9°), and for older than 80 years of age, 5.1° (3.5°, 6.85°). Overall, the median PUDA was 4.7°, with a first quartile of 2.8° and a third quartile of 6.5°, with an interquartile range of 3.7°.

These values were obtained using the combined measurements of both the initial and repeat measurements for the three independent observers. A Cronbach's alpha of 0.879 was obtained as the overall inter-rater reliability. For each individual observer, we obtained values of 0.871, 0.918, and 0.935 for our Cronbach's alpha for intra-rater reliability.

Multivariate regression analysis of demographic variables indicated that these variables did not substantially contribute to patient variance of the PUDA (adjusted R2 = 0.02, F (6, 508) = 2.704, *P* = 0.01). It was found that age was a significant predictor for the PUDA (β = 0.01, *P* = 0.02), as was height (β = 0.03, *P* = 0.02). The variables of BMI (*P* = 0.67), sex (*P* = 0.96), race (*P* = 0.06), and weight (*P* = 0.65) were not found to be significant predictors for the PUDA.

Approximately 97.4% of all elbows that had a PUDA of <5° accommodated the MUTARS implant; compared with 91.6% of the elbows with a PUDA ≥5° (*P* = 0.26). Regarding the SES ulnar stem component, we noted that the largest SES combination fit 100% of elbows with a PUDA ≤10° compared with 93% of elbows with a PUDA of >10° (*P* = 0.029). Elbows in which the largest SES combination fit were associated with a smaller PUDA whereas 97.4% of all elbows that had a PUDA of <5° accommodated the MUTARS implant; compared with 91.6% of the elbows with a PUDA ≥5° (*P* = 0.26). Regarding the SES ulnar stem component, we noted that the largest SES combination fit 100% of elbows with a PUDA ≤10° compared with 93% of elbows with a PUDA of >10° (*P* = 0.029). Elbows in which the largest SES combination fit were associated with a smaller PUDA when compared with elbows in which smaller SES combinations fit (5.4° versus 11.7°, *P* = 0.034). We also noted that elbows in which we established a fit with the MUTARS implant tended to have a smaller median PUDA when compared with elbows in which the MUTARS implant did not fit (5.4° versus 5.8°, *P* = 0.34) (Table 2).

**Table 2:** Templating of Ulnar Component Prostheses and Fit of Systems Across Angle Groups

Prosthetic System	Angle Group, <5°	Angle Group, 5–10°	Angle Group, >10°	*P*	PUDA (°), Median (Q1, Q3)	*P*
MUTARS, n (%)						
Fit	75 (97.4)	97 (91.5)	35 (92.1)	0.26^a^	5.4 (1.7, 9.1)	0.34^b^
No fit	2 (2.6)	9 (8.5)	3 (7.9)		5.8 (5, 9.8)	
SES, n (%)						
Large	77 (100)	106 (100)	36 (94.7)	0.029^a^	5.4 (1.8, 8.9)	0.034^b^
Other	—	—	2 (5.3)		11.7 (10.5, 12.8)	

MUTARS = Modular Universal Tumor and Revision System, PUDA = proximal ulna dorsal angulation, SES = Solar Elbow System

a Using the Fisher exact test.

b Using the Mann-Whitney *U* test.

## Discussion

To the best of our knowledge, this is the first study using the PUDA as an objective, preoperative measurement for templating of prosthetic elbow reconstruction. This measurement is easily obtained and is a valuable metric to minimize intraoperative complications. Although this study is conducted retrospectively and validated on templating software, we do feel this promotes an important framework for consideration of ulnar component stem fit in elbow reconstruction. Future prospective studies would be useful in validating the benefit of the PUDA in surgical practice, but we believe the findings here represent an important first step in establishing the principle of decreasing PUDA measurement correlating with larger stem fit. In addition, future studies may take into account other factors affecting bone morphology or growth, including childhood or genetic syndromes, bone densitometric data, or other endocrinological previous conditions that may have affected development of the bony skeleton before skeletal maturity. We also concede that in cases of pathologic fracture, it may be more difficult to accurately use the PUDA in preparation for elbow reconstruction, although in these scenarios, elbow radiographs of the contralateral elbow may be of use.

This study is also novel in that although demographic factors such as ethnicity, sex, and age have been shown in multiple studies to influence bone density as well as bone geometry,^12,13,14,15,16,17,18,19^ few studies have assessed the geometric anatomy of the olecranon with respect to the dorsal ulnar cortex or have described the influence of these demographic factors on the PUDA. Puchwein et al^12^ found that preshaped plates for fixation of comminuted and Monteggia fractures of the ulna had large variability, ranging from 23% to 88%, in achieving an appropriate fit. This suggests that the preoperative step of templating reconstruction systems and measurement of the PUDA can be important in accessing appropriate fit.

We noted several important findings from our study as they pertain to elbow replacement systems. First, we noted that the curved ulnar stem of the MUTARS system was a limiting factor in the ability of our templated elbows with the PUDA ≥5° to accommodate the ulnar component. As such, in patients with a PUDA of ≥5°, alternatives to the MUTARS should be made available as the curved ulnar stem may not fit. Production of several size selections for the curved ulnar stem may also be useful as straight ulnar stems are smaller in general, making them prone to loosening or stem fracture. Regarding the SES components, we similarly noted that elbows with a PUDA >10° required a combination of either a smaller stem length or stem diameter, which corroborates our findings with the MUTARS ulnar stem component. This makes intuitive sense, as a larger angle between the olecranon and ulnar canal would be expected to have a smaller canal width overlap, requiring either a more curved component or a smaller combination of stem length and/or diameter in the case where the angle of curvature of the stem does not equal the curvature of the traversed canal path going from the olecranon into the ulnar canal. In these situations, ulnar component fixation may thus be more likely to fail due to a high stem to intramedullary space ratio. These situations may be aided by a larger osteotomy of the proximal olecranon to permit a more straightforward insertion trajectory of the stem into the ulnar diaphysis.

This study had several limitations. Multiple reviewers for the lateral elbow radiographs introduce the potential effects of bias in measurement as well as the possibility of poor inter-rater and intra-rater reliability. To minimize this effect, we blinded each reviewer to the angle measurements of the other reviewers as well as their previous set of measurements in their subsequent review 3 months later. We do believe this scenario is a more realistic representation of clinical practice. In addition, although perfect lateral views were attempted in each case, there is likely to be some unavoidable variation and likely obliquity in some of the radiographs. To control for this, however, we did strive to robustly review the technical adequacy of the lateral elbow radiographs and specifically made this an exclusion criterion in our study; indeed, lateral review adequacy was determined qualitatively on review of flagged radiographs. To minimize confounding in this regard, in cases where one reviewer deemed a radiograph inadequate, that radiograph was discarded for the other two reviewers. Finally, magnification markers were not on the radiographs which unfortunately were not able to be added given the retrospective nature of the study. However, to minimize potential confounding in this regard, each radiograph was reviewed individually and qualitatively by trained personnel in orthopaedic surgery or radiology. In addition, these data were collected from contiguous radiographs to further minimize radiographic technician turnover and taken from radiographs conducted using the same radiograph machine with the same radiographic technician staff, and as such, we feel more reassured that there is no suggestion from review of these radiographs that calibration was not attained. Finally, templating was conducted by the same individual throughout the manuscript to minimize heterogeneity in measurements, such that any trends noted would remain internally consistent.

Another limitation to our study was the random selection of 221 radiographs from each age bracket. This may have limited our ability to establish statistical significance in our analyses of ulnar stem component sizing. Of note, we introduced confounding effect when we templated all 514 elbows, as we had disproportionately low representation of elbows from the largest angle category. Although we did not conduct an analysis of the optimal number of elbows that could be templated while still recapitulating our observed effects noted during the templating process, we did conduct several successive rounds of templating using higher and higher numbers of templated elbows until we noted faithful recapitulation of effect without excessive confounding from disproportionate representation between angle groups which may have diluted the significance of our findings with the MUTARS system. We did, however, encouragingly note that these findings nonetheless recapitulated the trend we observed in the SES system, where we observed the statistically significant finding that the largest SES combination required a smaller PUDA when compared with other SES combinations.

On a more fundamental level, the retrospective nature of this study may limit some of the applicability of this data set; it would be invaluable to perform future studies concerning the PUDA in a prospective manner with the opportunity to validate these measurements intraoperatively. In addition, the study was restricted to two modular elbow reconstruction systems by the availability of templating software. We attempted to include other systems to broaden the applicability of our findings, but no templating software was available either from these companies or from templating software developers despite multiple rounds of contact with representatives from these companies; this lack of templating software precluded our ability to validate our measurements in other systems. Future studies should validate these findings intraoperatively in patients receiving surgical reconstruction using these systems as well as any available additional systems.

Despite these limitations, we believe that the PUDA should be measured in preoperative planning for sizing the ulnar stem component in distal humeral replacement to ensure intraoperative fit and reduce complications. Indeed, even robust preoperative review of lateral radiographs can result in overestimation of the minimal canal size of the ulna in selecting the ulnar component, predisposing to complications.

We describe the PUDA as a valuable preoperative planning tool for prosthetic elbow reconstruction after distal humeral tumor resection, which is more easily accessible and faster than templating, and may be additionally useful in situations where templating software is not available. The median PUDA obtained in this study is comparable to values obtained in other studies; our study is the largest cohort of patients with measured values of the PUDA to our knowledge in the literature.^10-12^ Based on our findings, commonly available demographic data do not substantially contribute to the PUDA measurement, necessitating preoperative measurement of the PUDA on lateral elbow radiographs in selecting an ulnar stem component size. Attentive planning and careful prosthetic selection should be exercised in all patients, but particularly so in those with an PUDA measurement greater than 5°, where a smaller ulnar component stem should be considered.
